# A single dose of cannabidiol modulates medial temporal and striatal function during fear processing in people at clinical high risk for psychosis

**DOI:** 10.1038/s41398-020-0862-2

**Published:** 2020-09-13

**Authors:** Cathy Davies, Robin Wilson, Elizabeth Appiah-Kusi, Grace Blest-Hopley, Michael Brammer, Jesus Perez, Robin M. Murray, Paul Allen, Matthijs G. Bossong, Philip McGuire, Sagnik Bhattacharyya

**Affiliations:** 1 Department of Psychosis Studies, Institute of Psychiatry, Psychology & Neuroscience, King’s College London, London, UK; 2 Department of Neuroimaging, Institute of Psychiatry, Psychology & Neuroscience, King’s College London, London, UK; 3 CAMEO Early Intervention Service, Cambridgeshire and Peterborough NHS Foundation Trust, Cambridge, UK; 4 Department of Psychology, University of Roehampton, London, UK; 5 Icahn School of Medicine, Mount Sinai Hospital, New York, NY USA; 6 Department of Psychiatry, University Medical Center Utrecht Brain Center, Utrecht University, Utrecht, The Netherlands; 7 National Institute for Health Research (NIHR) Maudsley Biomedical Research Centre (BRC), South London and Maudsley NHS Foundation Trust, London, UK; 8 Outreach And Support in South London (OASIS) Service, South London and Maudsley NHS Foundation Trust, London, UK

**Keywords:** Neuroscience, Schizophrenia

## Abstract

Emotional dysregulation and anxiety are common in people at clinical high risk for psychosis (CHR) and are associated with altered neural responses to emotional stimuli in the striatum and medial temporal lobe. Using a randomised, double-blind, parallel-group design, 33 CHR patients were randomised to a single oral dose of CBD (600 mg) or placebo. Healthy controls (*n* = 19) were studied under identical conditions but did not receive any drug. Participants were scanned with functional magnetic resonance imaging (fMRI) during a fearful face-processing paradigm. Activation related to the CHR state and to the effects of CBD was examined using a region-of-interest approach. During fear processing, CHR participants receiving placebo (*n* = 15) showed greater activation than controls (*n* = 19) in the parahippocampal gyrus but less activation in the striatum. Within these regions, activation in the CHR group that received CBD (*n* = 15) was intermediate between that of the CHR placebo and control groups. These findings suggest that in CHR patients, CBD modulates brain function in regions implicated in psychosis risk and emotion processing. These findings are similar to those previously evident using a memory paradigm, suggesting that the effects of CBD on medial temporal and striatal function may be task independent.

## Introduction

There are currently no licensed clinical interventions for people at clinical high risk for psychosis (CHR)^1,2^. One of the most promising candidate treatments is cannabidiol (CBD), a phytocannabinoid constituent of the cannabis plant^3^. While the main psychoactive cannabinoid in cannabis, delta-9-tetrahydrocannabinol (THC), has psychotomimetic^4–7^ and potential anxiogenic effects, CBD is non-intoxicating and has both anxiolytic^8,9^ and antipsychotic properties^10–12^. However, the neural mechanisms of action that underlie these effects are still unclear. In healthy volunteers, CBD modulates neural responses to cognitive and emotional tasks in several regions, particularly the medial temporal cortex and the striatum, as well as functional connectivity between these regions^13–18^. Similarly, in clinical samples, CBD has been shown to modulate activation and functional connectivity between medial temporal cortex and striatum during verbal memory processing in people at CHR^19^ and those with established psychosis^20^. Effects in these regions are of particular interest as they are critically implicated in the onset of psychosis^21–26^. However, whether the effects of CBD on the medial temporal cortex and striatum in CHR subjects are specific to verbal memory processing or are also evident in the context of other cognitive or emotional processes remains unclear.

Emotional dysregulation is a common feature of the CHR state and contributes to distress and to poor functional outcomes^27–31^. Evidence suggests that CHR subjects show altered neural responses to emotion (and particularly fear) processing stimuli in limbic and paralimbic regions (the hippocampus, parahippocampal gyrus and amygdala), striatum and frontal cortex^30,32,33^. Abnormal neurofunctional responses to emotional stimuli in these regions may also underlie the high levels of anxiety experienced by these patients and contribute to the generation of attenuated psychotic symptoms by fuelling aberrant salience^28,30,34–37^. CBD is known to have anxiolytic effects in both animals and man^10,38^; offline studies show that CBD reduces anxiety^39^ in people with social anxiety disorder^8,40^ and in healthy people subjected to experimental stress, such as simulated public speaking^41–43^ (reviewed in ref. ^10^). CBD also attenuates the anxiogenic effects of THC and modulates brain function in the opposite direction during fear processing^13,14,44^. For example, a previous study showed that the processing of fearful (relative to neutral) faces under placebo conditions is associated with activation in the parahippocampal gyrus and amygdala, and while THC induced physiological anxiety, CBD attenuated activation in these brain regions, which was associated with a reduction of physiological anxiety^13^. The anxiolytic properties of CBD are thus potentially mediated by its effects on the same brain regions that are altered in CHR patients.

The present study examined the effects of CBD on regional brain activation in CHR subjects while they viewed faces with fearful (vs neutral) expressions. On the basis of data from previous studies (above), the two primary regions of interest (ROIs) were the medial temporal lobe (hippocampus, parahippocampal gyrus and amygdala) and the striatum/pallidum (caudate, putamen and globus pallidus). These regions are known substrates of emotion (and particularly fear) processing^45–47^ and this task has previously been shown to engage these processes and brain regions^13^. We first hypothesised that, relative to healthy controls, CHR patients under placebo conditions would show altered engagement of the medial temporal lobe and striatum during fear processing. Our second hypothesis was that CHR patients receiving CBD would then show a ‘normalisation’ of activation in the same regions identified as differentially engaged in the placebo vs control analyses. That is, activation in the CBD group would be intermediate between that observed in the healthy control and CHR placebo groups.

## Patients and methods

### Participants

The study received Research Ethics (Camberwell St Giles) approval and all participants provided written informed consent. Thirty-three antipsychotic-naive CHR individuals, aged 18–35 years, were recruited from specialist early detection services in the United Kingdom. CHR status was determined using the Comprehensive Assessment of At-Risk Mental States (CAARMS) criteria^48^. Briefly, subjects met one or more of the following subgroup criteria: (a) attenuated psychotic symptoms, (b) brief limited intermittent psychotic symptoms (psychotic episode lasting <1 week, remitting without treatment), or (c) either schizotypal personality disorder or first-degree relative with psychosis, all coupled with functional decline^48^. Nineteen age- (within 3 years), sex- and ethnicity-matched healthy controls were recruited locally by advertisement. Exclusion criteria included history of psychotic or manic episode, current Diagnostic and Statistical Manual of Mental Disorders, Fourth Edition diagnosis of substance dependence (except cannabis), intelligence quotient <70, neurological disorder or severe intercurrent illness and any contraindication to magnetic resonance imaging (MRI) or treatment with CBD. Inclusion/exclusion criteria were pre-specified. Participants were required to abstain from cannabis for 96 h, other recreational substances for 2 weeks, alcohol for 24 h and caffeine and nicotine for 6 h before attending. A urine sample prior to scanning was used to screen for illicit drug use and pregnancy.

### Design, materials, procedure

The study was registered (ISRCTN.org identifier: ISRCTN46322781) and the protocol (including power calculation) has been previously published (supplement in ref. ^19^).

Using a randomised, double-blind, placebo-controlled, three-arm, parallel-group design, CHR participants were randomised to a single oral 600 mg dose of CBD (THC-Pharm, Germany) or a matched placebo capsule. ﻿This dose was selected based on previous findings that doses of 600–800 mg/day are effective in established psychosis^11^ and anxiety^8,10,49^. Psychopathology was measured at baseline (before drug administration) using the CAARMS (positive and negative symptoms) and State-Trait Anxiety Inventory (State Subscale). Following a standard light breakfast, participants were administered the capsule (at ~11 a.m.) and, 180 min later, underwent functional MRI (fMRI) while performing a fearful faces task. This interval between drug administration and fMRI acquisition was selected based on previous findings describing peak plasma concentrations at 180 min following oral administration^50,51^. Control participants were investigated under identical conditions but did not receive any study drug. Plasma CBD levels were sampled at baseline (before taking the study drug) and at 120 and 300 min after drug administration.

### Functional MRI

#### Image acquisition

All scans were acquired on a General Electric Signa HDx 3 T MR system. Functional images were acquired using echo planar imaging (EPI) with parameters: repetition time (TR) = 2000 ms, echo time (TE) = 30 ms, flip angle = 75°, 39 × 3 mm slices, 3.3 mm slice gap, matrix = 64 × 64, field of view (FoV) = 240, 180 timepoints. T1-weighted structural images (inversion recovery EPI; TE = 30 ms, TR = 3000 ms, 43 × 3 mm slices, FoV = 240 mm, matrix = 128 × 128) were also acquired for co-registration.

#### fMRI task

Participants were studied in one 6-min fMRI experiment while performing a fearful face processing task (described in detail elsewhere^13,14,52^). In short, the blood-oxygen-level-dependent (BOLD) haemodynamic response was measured using an event-related design while subjects viewed fearful faces (mild fear, intense fear), which were contrasted with faces with neutral expressions. Ten different facial identities each conveying a neutral, mild fear and intense fear expression (30 different facial stimuli) were presented twice each for 2 s, resulting in 60 facial stimuli in total. The order of presentation of facial identities and expression type was pseudorandomised such that the same identity or expression type was not presented in successive trials. The inter-trial interval was varied from 3 to 8 s according to a Poisson distribution, with an average interval of 4.9 s. A fixation cross was presented during the inter-stimulus interval. Participants were asked to indicate the gender of the face via button press, with the speed and accuracy of responses recorded online throughout image acquisition.

### Analysis

fMRI data were analysed with the XBAM software v4.1 using a nonparametric approach to minimise assumptions^53,54^. For each group (control, placebo, CBD), we contrasted the active task condition (mild and intensely fearful faces) against the baseline condition (neutral faces) to identify the brain regions engaged by the processing of fear after controlling for activation related to face processing independent of emotional expression.

Images were corrected for motion^55^ and smoothed with a 5-mm Gaussian filter. Individual activation maps were created using two γ-variate functions to model the BOLD response^56^. Following a least-squares fitting of this model, the sum of squares (SSQ) ratio statistic (ratio of the model component to the residual sum of squares) was estimated at each voxel, followed by permutation testing to determine significantly activated voxels specific to each condition (neutral, mild fear, intense fear)^57,58^. SSQ ratio maps for each individual were transformed into standard stereotactic space^54,59^. Group activation maps for each condition (and then for neutral vs mild fear and neutral vs intense fear) were computed for each group (control, CBD, placebo) by determining the median SSQ ratio at each voxel (over all individuals). Mild and intense fear were thereafter analysed as a single fearful faces condition. Group activation maps for fearful vs neutral conditions were compared between participant groups (placebo vs control) or treatment conditions (CBD vs placebo) using nonparametric analysis of variance (ANOVA)^53^ and an ROI approach. A single ROI mask was constructed using the Talairach atlas daemon, which included the bilateral medial temporal lobe (hippocampus, parahippocampal gyrus and amygdala) and the striatum/pallidum (caudate, putamen and globus pallidus). These regions were selected a priori based on our previous findings^19^. The voxel-wise statistical threshold was set at *p* = 0.05, and the cluster-wise thresholds were adjusted to ensure that the number of false-positive clusters per brain would be <1; clusters that survived this critical statistical threshold and the corresponding *p* values are reported.

In line with our first hypothesis, we first compared the placebo-treated CHR group with healthy controls to identify areas (within our pre-defined ROI network) showing altered activation related to the CHR state. We then directly compared CHR patients under placebo with those under CBD (within the same pre-defined ROI network) to test whether CBD had effects on the same brain regions that were identified as having altered activation associated with CHR status (as in the comparison of placebo-treated CHR participants with healthy controls above). Finally, to test the hypothesis that activation in the CBD group would be intermediate between that of the control and ﻿placebo groups, we examined whether a linear relationship in brain activation (placebo group > CBD group > control group or placebo group < CBD group < control group) existed within the same ROI network.

#### Behavioural task analyses

All non-imaging data were analysed using SPSS 24. Extreme values (>3 × interquartile range identified in boxplots) within the behavioural task data were excluded from task performance analyses. The percentage of correct responses and reaction times were analysed using mixed ANOVAs, with group (control, placebo, CBD) as the between-subject factor and emotional valence (neutral, fearful) as the within-subject factor. Robustness of findings to outliers was tested using sensitivity analyses. Significance was set at *p* < 0.05.

## Results

There were no between-group differences in the majority of demographic and baseline clinical characteristics, except for fewer years of education in the placebo group relative to controls (Table 1). ﻿In the CBD group, mean plasma CBD levels were 126.4 nM (SD = 221.8) and 823.0 nM (SD = 881.5) at 120 and 300 min after drug intake, respectively. Three CHR individuals exited the scanner prior to the fMRI task, leaving 15 subjects in the placebo group, 15 in the CBD group and 19 healthy controls.

**Table 1:** Sociodemographic and clinical characteristics at baseline.

Characteristic	CBD (*n* = 16)	Placebo (*n* = 17)	Control (*n* = 19)	Pairwise comparison	
				Control vs placebo	Placebo vs CBD
Age, years; mean (SD)	22.7 (5.08)	24.1 (4.48)	23.9 (4.15)	*p* = 0.91^a^	*p* = 0.42^a^
Sex, *N* (%) male	10 (62.5)	7 (41.2)	11 (57.9)	*p* = 0.32^b^	*p* = 0.22^b^
Ethnicity, *N* (%)					
White	10 (62.5)	7 (41.2)	11 (57.9)	*p* = 0.59^b^	*p* = 0.43^b^
Black	2 (12.5)	5 (29.4)	5 (26.3)		
Asian	0 (0)	1 (5.9)	0 (0)		
Mixed	4 (25)	4 (23.5)	3 (15.8)		
Education, years; mean (SD)	14.4 (2.71)	12.6 (2.76)	16.9 (1.58)	***p*** < **0.001**^a^	*p* = 0.06^a^
CAARMS score, mean (SD)					
Positive symptoms	40.19 (20.80)	42.94 (29.47)	NA	NA	*p* = 0.76^a^
Negative symptoms	23.25 (16.49)	28.41 (20.49)	NA	NA	*p* = 0.43^a^
STAI-S, mean (SD)	40.31 (9.07)	38.94 (10.18)	NA	NA	*p* = 0.69^a^
Urine drug screen results, *N* (%)					
Clean	10 (63)	8 (47)	0 (0)	NA^c^	*p* = 0.45^b^
THC	2 (13)	5 (29)	0 (0)		
Morphine	1 (6)	0 (0)	0 (0)		
Benzodiazepines	0 (0)	1 (6)	0 (0)		
PCP	0 (0)	1 (6)	0 (0)		
Missing	3 (19)	2 (12)	0 (0)		
Current nicotine use, *N* (%) yes	9 (56.3)	5 (29.4)	2 (10.5)	*p* = 0.15^b^	*p* = 0.12^b^
Current cannabis use, *N* (%) yes	7 (43.8)	7 (41.2)	0 (0)^d^	NA^c^	*p* = 0.88^b^
Handedness, *N* (%) right	14 (87.5)	17 (100)	18 (94.7)	*p* = 0.37^b^	*p* = 0.16^b^

*CAARMS* Comprehensive Assessment of At-Risk Mental States, *CBD* cannabidiol, *CHR* clinical high risk for psychosis, *N* number of subjects, *NA* not applicable, *PCP* phencyclidine, *STAI-S* State-Trait Anxiety Inventory-State Subscale, *THC* Δ9-tetrahydrocannabinol.

^a^Independent *t* test.

^b^Pearson chi-squared test.

^c^Controls were selected to have minimal drug use and hence were not compared with CHR participants on these parameters.

^d^Cannabis use <10 times lifetime (no current users).

Bold text indicates significant difference.

### Task performance

#### Accuracy

One subject from each group had no useable offline task data and one healthy control was removed due to extreme low task performance (gender discrimination accuracy) values, leaving 14 participants in the placebo group, 14 in the CBD group and 17 controls for task accuracy and reaction time analyses. Subjects distinguished the gender of faces with a percentage mean ± SD accuracy of 87.94 ± 2.25 in controls, 88.33 ± 2.61 in the placebo group and 86.07 ± 3.96 in the CBD group. There was no main effect of group, valence or a group × valence interaction on task performance (all *p* > 0.05). Removal of outliers made no material change to the results.

#### Reaction times

Across all individuals, there was a significant main effect of valence (*F*(1,43) = 8.47, *p* = 0.006) with subjects responding significantly faster (in gender discrimination) to fearful relative to neutral faces. There was no main effect of group (*F*(2,43) = 2.71, *p* = 0.078) and no interaction between group and valence (*F*(2,43) = 2.09, *p* = 0.137). After removal of one potential outlier, the main effect of group became significant (*F*(2,42) = 4.96, *p* = 0.012), with healthy controls responding significantly faster than the CBD group.

### fMRI results

#### Task network in healthy controls

In healthy controls, decreased activation was observed in the left parahippocampal gyrus during the processing of fearful relative to neutral faces (peak Talairach coordinates *x* = −25, *y* = −41, *z* = −7; *k* = 17; *p* < 0.001). There were no significant effects in the opposite direction (fearful > neutral faces).

#### Differences in activation associated with the CHR state (placebo vs controls)

**Fig. 1:**
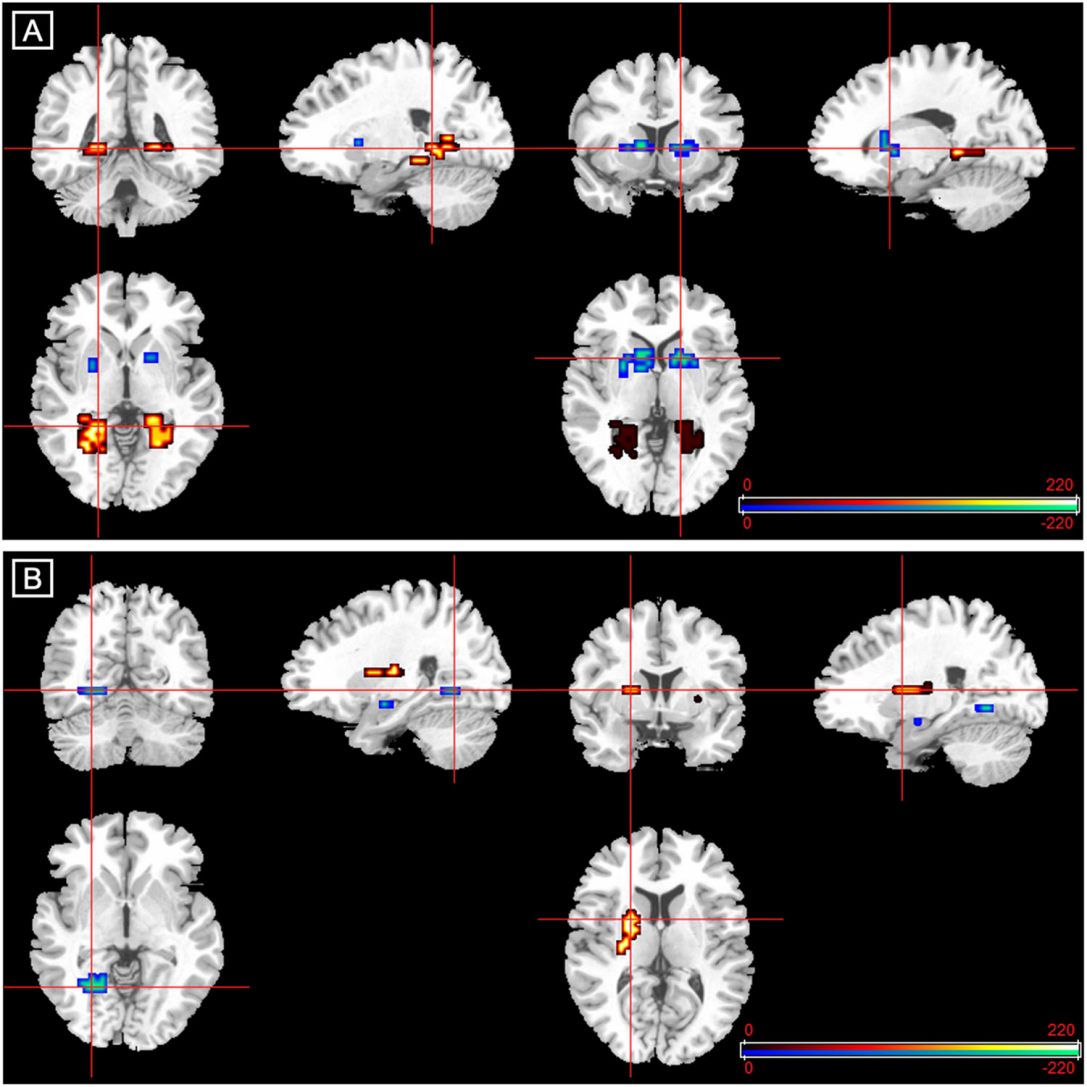
Altered brain activation in participants at clinical high risk of psychosis (CHR) and effect of cannabidiol (CBD). **a** Fear processing in the CHR-placebo vs control group. Clusters showing greater (red/yellow) or reduced (blue/green) activation in participants at clinical high risk receiving placebo compared with healthy controls during fear processing. **b** Fear processing in the CHR-CBD vs CHR-placebo group. Clusters showing greater (red/yellow) or reduced (blue/green) activation in participants at clinical high risk receiving cannabidiol (CBD) compared with those receiving placebo during fear processing. The right side of the brain is shown on the right of the images.

During the processing of fearful relative to neutral faces, compared to healthy controls, CHR subjects receiving placebo showed augmented activation in the left lingual gyrus and bilateral parahippocampal gyri and attenuated activation in the striatum bilaterally, including the left caudate head and putamen, the right putamen and a smaller cluster in the right caudate head (Table 2 and Fig. 1).

**Table 2:** Differences in activation between 15 participants at clinical high risk for psychosis (CHR) receiving placebo, 19 healthy controls and 15 CHR participants receiving cannabidiol (CBD).

Region	Talairach coordinates			Cluster size, no. of voxels	*p* Value^a^
	*x*	*y*	*z*		
**Differences between healthy controls and CHR-placebo**					
Placebo > controls					
Parahippocampal gyrus	−18	−33	−13	5	0.002
Parahippocampal gyrus	−25	−44	−7	35	<0.001
Parahippocampal gyrus	18	−33	−3	22	<0.001
Lingual gyrus	−22	−56	3	5	0.003
Controls > placebo					
Putamen	−22	11	0	5	0.002
Caudate head	−11	19	0	10	<0.001
Putamen	25	11	3	7	0.001
Putamen	22	15	0	13	<0.001
**Differences between CHR-placebo and CHR-CBD**					
Placebo > CBD					
Amygdala	−25	−4	−16	4	0.002
Parahippocampal gyrus	−18	−56	−7	11	<0.001
CBD > placebo					
Putamen	25	15	0	6	0.001
Putamen	−18	11	7	16	<0.001

^a^Corrected for <1 false-positive cluster.

#### Effects of CBD on activation in participants at CHR (CBD vs placebo)

During fear processing, compared to CHR participants receiving placebo, those in the CBD group showed lower activation in the left parahippocampal gyrus and in a small cluster in the left amygdala and greater activation in the left putamen and in the right putamen extending to the caudate head (Table 2 and Fig. 1).

#### Between-group linear analysis

**Fig. 2:**
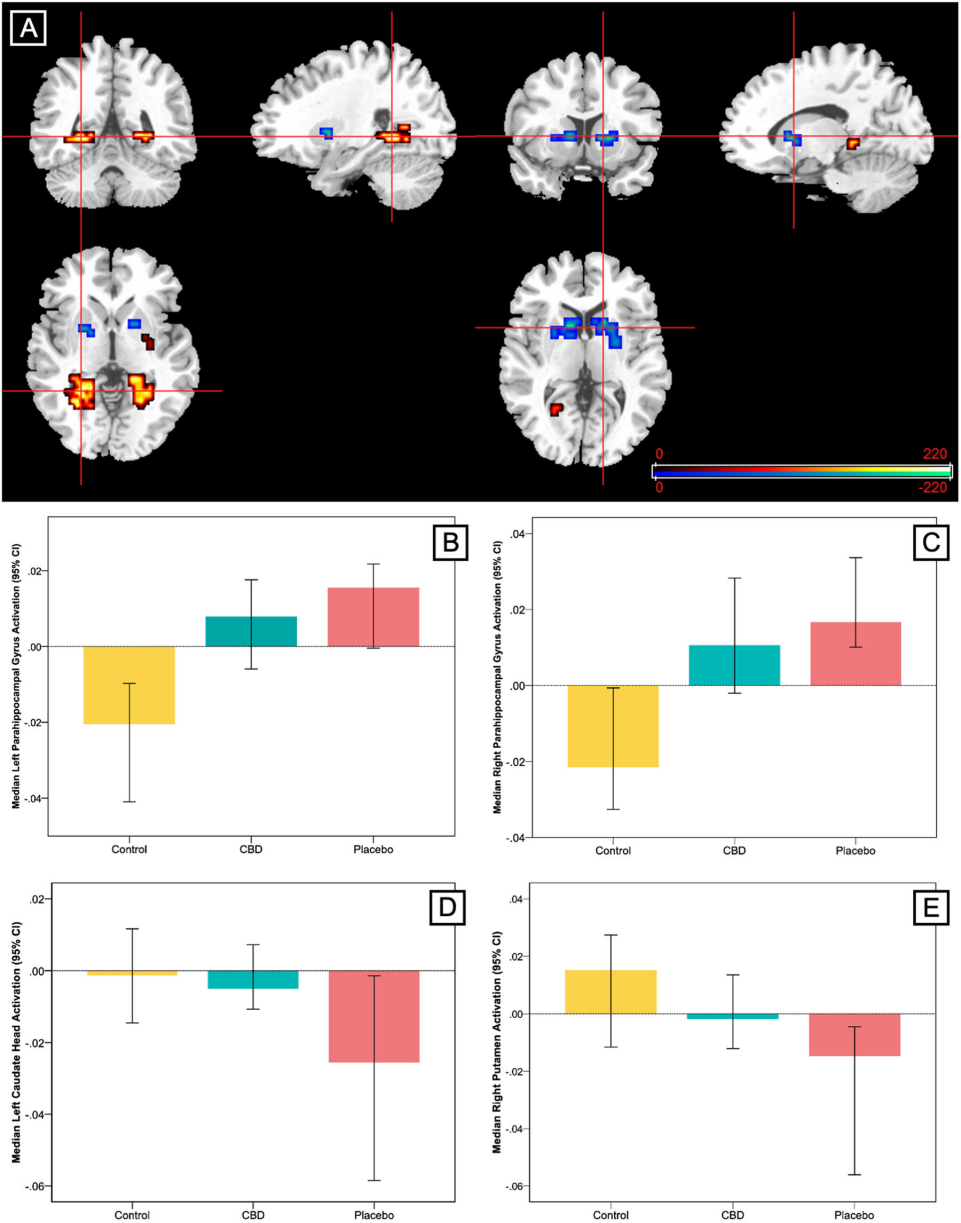
Effect of cannabidiol (CBD) on brain activation compared with placebo in participants at clinical high risk of psychosis (CHR) and healthy control participants. **a** Clusters where activation differed across the three groups in a linear relationship during fear processing. In the parahippocampal region (red/yellow), activation was greatest in the group of clinical high risk participants receiving placebo, lowest in healthy controls and intermediate in the CBD group. In the striatum (blue/green), activation was greatest in healthy controls, lowest in participants at clinical high risk receiving placebo and intermediate in participants at clinical high risk receiving CBD. The right side of the brain is shown on the right of the images. **b**–**e** Median activation in each group in **b** the left parahippocampal gyrus, **c** the right parahippocampal gyrus, **d** left caudate head and **e** right putamen during fear processing in arbitrary units as indexed using the median sum of squares ratio. The sum of squares ratio statistic refers to the ratio of the sum of squares of deviations from the mean image intensity due to the model (over the whole time series) to the sum of squares of deviations due to the residuals.

This analysis identified clusters where the pattern of regional brain activation during fear processing showed a linear relationship across the three groups, such that activation in the CBD group was intermediate to that of the placebo and control groups. A linear relationship was observed in relatively large clusters in the bilateral parahippocampal gyri, with the greatest activation in the group of CHR participants receiving placebo, the lowest in healthy controls and intermediate activation in the CBD group (Table 3 and Fig. 2). These clusters directly overlapped with the parahippocampal clusters differentially engaged by the control and placebo groups in the two-group analyses. The opposite linear pattern was observed in the striatum. Here the highest level of activation was found in healthy controls, the lowest in CHR participants receiving placebo and intermediate activation in the CBD group (Table 3 and Fig. 2). Again, these clusters directly overlapped with the clusters found to be differentially engaged in the placebo vs healthy control group analyses. Removal of the healthy control subject with extreme low task performance (accuracy) scores made no material change to the imaging results (data not shown here).

**Table 3:** Linear relationship in activation across 15 participants at clinical high risk for psychosis (CHR) receiving placebo, 19 healthy controls and 15 CHR participants receiving cannabidiol (CBD).

Region	Talairach coordinates				Cluster size, no. of voxels	*p* Value^a^
	*x*	*y*		*z*		
Placebo > CBD > controls						
Parahippocampal gyrus	−25	−44	−7		37	<0.001
Parahippocampal gyrus	18	−33	−3		25	<0.001
Controls > CBD > placebo						
Putamen	−18	7		−3	5	0.001
Caudate head	−7	19		0	10	<0.001
Putamen	22	15		0	11	<0.001
Putamen	25	4		3	8	0.001

^a^Corrected for <1 false-positive cluster.

## Discussion

We investigated differences in brain function during fear processing between CHR subjects and healthy controls and examined the effects of a single dose of CBD. As expected, relative to healthy controls, CHR individuals under placebo conditions showed attenuated striatal and augmented parahippocampal activation during fear processing. The major finding of the present study was that, as predicted, a single dose of CBD modulated activation in these regions such that activation in the CHR subjects given CBD was intermediate to that observed in CHR subjects given placebo and the healthy controls.

These results are broadly consistent with those from a previous study^19^, wherein we examined the same individuals under identical conditions, except that activation was measured during a verbal memory task, rather than an emotional processing task. In both studies, we find that CBD modulated parahippocampal and striatal activation^19^. Moreover, the direction of the effects of CBD in both studies were such that they reflected a normalisation of the dysfunction observed in the respective CHR-placebo vs control group analyses. CBD has also been found to attenuate dysfunction of mediotemporal activation and mediotemporal–striatal functional connectivity during memory processing in patients with first-episode psychosis^20^. Taken together, the data from the present study extends previous results to suggest that the acute effects of CBD on activation in the medial temporal cortex and striatum, key brain regions implicated in the onset of psychosis^22–24^, may be task independent. However, the precise direction of effects of CBD in these regions differ between the two CHR studies (further discussed below).

Previously, during a verbal memory task, we found that CHR individuals under placebo conditions showed less activation in the caudate (during encoding) and in the parahippocampal gyrus (during recall) compared to controls, and CBD augmented activation in both regions^19^. In contrast, during fear processing in the present study, CHR individuals showed reduced activation in the striatum and enhanced activation in the parahippocampal gyri compared to controls, and CBD attenuated parahippocampal activation while augmenting striatal activation. The primary between-study difference in the direction of CBD effects therefore appears in the medial temporal lobe, which may be accounted for by the differential role of this region in verbal memory vs fear processing paradigms. In verbal memory processing, the parahippocampal gyrus is involved in the binding of contextual and relational information to support memory encoding and recall^60,61^. Recall performance was found to be correlated with parahippocampal engagement^19^, suggesting that in the context of pathology/insufficient recruitment of this region to meet mnemonic demands, CBD may act to optimise parahippocampal engagement. This accords with the finding that CBD protects verbal memory against the detrimental effects of THC^6^ and partially normalises aberrant brain function during memory processing in first-episode psychosis^20^. Conversely, during fear processing, the parahippocampal gyrus and amygdala are known to activate in response to fear/threat-related environmental cues, particularly angry or fearful facial stimuli^45–47^. In the current study, both parahippocampal and amygdala activation were attenuated by CBD, suggesting that CBD may partially normalise (attenuate) the altered neurofunctional response to fear/threat-related stimuli in CHR patients, which is in line with the potential anxiolytic effects of CBD and the role of the endocannabinoid system as a regulator of subjective affective states, including anxiety, fear and aggression^62–64^. Indeed, previous work has shown that CBD attenuates limbic and paralimbic function in healthy individuals^13,65^ and in patients with anxiety disorders^9^, and this is related to its anxiolytic effects^9,13^. In terms of more general anxiolytic effects, offline studies show that CBD reduces anxiety^39^ in people with social anxiety disorder^8,40^ and in healthy people subjected to experimental stress, such as simulated public speaking^41–43^ (reviewed in ref. ^10^). Consistent with this, we recently found that a short (7 day) course of CBD treatment partially attenuated abnormal neuroendocrine (cortisol) and psychological (anxiety and stress perception) responses to experimentally induced social stress in CHR patients^66^. Together, these findings support further research into the potential utility of CBD for ameliorating anxiety both within and outside of CHR populations. Whether the effects of CBD in CHR individuals arise through the specific targeting of psychosis-related pathophysiology or are due to more generic effects (for instance, on state anxiety) remains an important avenue for future research.

Our findings also agree with what is known about the opposite effects of THC and CBD on emotion-processing-related circuitry in healthy people. In the majority of (but not all^67,68^) studies, THC appears to augment amygdala activation and increase anxiety during fearful face processing^14,62^ and reduces amygdala–prefrontal connectivity during negative affect reappraisal^62^. Conversely, CBD increases fronto-striatal connectivity^69^ and attenuates amygdala activation while concomitantly decreasing physiological anxiety^13,14^. Some (but not all^70^) offline studies also show that CBD improves emotional face recognition while THC impairs it, and combining CBD with THC prevents the impairing effects of THC^71^.

The finding that CHR patients show alterations in brain function during fear processing is consistent with previous work showing dysfunction in medial temporal and striatal regions in CHR individuals across numerous cognitive paradigms^19,72–74^, as well as evidence of elevated limbic response in those with psychosis-spectrum features^35^ and individuals at genetic risk^75^, and altered amygdala/hippocampal activation in those with established psychosis^76,77^. Meta-analyses of >100 fMRI data sets indicate that the parahippocampal gyrus is active during the processing of emotional faces^46^, and emotion (particularly fear) processing in humans is associated with increased dopamine neurotransmission in the parahippocampal gyrus and striatum^47^. Enhanced parahippocampal activation in CHR individuals in the present study may therefore reflect an overactivation to emotional stimuli, in keeping with the notion that hippocampal hyperactivation is critical to psychosis onset^21–23,26^, and is consistent with previous evidence of elevated limbic response in those with psychosis-spectrum features^35^ and individuals at genetic risk^75^. The enhanced activation in the current study may also reflect a failure to deactivate limbic and paralimbic regions after repeated presentations of fear/threat-related stimuli^78,79^, as has been suggested^80^.

Attenuated activation in the striatum in CHR individuals may reflect disrupted emotional salience processing. A study of emotional prosodic voice recognition found that in healthy controls the caudate was activated in response to negative (vs neutral) stimuli, whereas CHR individuals showed the opposite pattern: greater activation to neutral stimuli^32^. These findings echo further work showing that CHR individuals hyperactivate frontal and temporal regions in response to neutral (vs emotional) faces^33^, and greater corticolimbic activation to neutral (vs emotional) scenes is associated with higher levels of positive symptoms and poorer functioning in CHR patients^30^. This phenomenon is also observed in the hippocampus and amygdala in patients with established psychosis^81^. Conceptually, fearful facial stimuli are expected to be more salient than neutral (innocuous) stimuli. Misattribution of salience by CHR individuals in this context may underlie the deficits in recognising and interpreting the emotions and intentions of others^82,83^. This, in turn, may contribute to anxiety, paranoia and the development of attenuated psychotic symptoms^37^, which are characteristic of the CHR state.

While the present study and previous work points towards potential neurophysiological mechanisms underlying the antipsychotic and anxiolytic effects of CBD, the precise molecular mechanism(s) remain incompletely understood. Preclinical and in vitro work suggests that the effects of CBD may be mediated by various mechanisms, including negative allosteric modulation of the CB1 receptor^84^, inhibition of anandamide hydrolysis^85^, actions on 5-HT1A receptors^86^, vanilloid type 1 receptors^85^, GPR55 receptors^87,88^, modulation of the glutamate system^89^ and various other mechanisms^90,91^. Further preclinical evidence points to neuroprotective, antioxidant and anti-inflammatory properties of CBD^10^. However, direct evidence in humans is lacking. Although functional neuroimaging results are almost certainly downstream from primary molecular effects^87^, they offer crucial insight into the neural substrates and systems-level effects of CBD in vivo in target patient populations.

Our results should be considered in the context of certain limitations, one of which was the absence of a within-subject design. The possibility that between-group differences were attributable to between-subject variability, as opposed to an effect of CBD, cannot therefore be completely excluded. Because we used an ROI approach, focussing on the striatum/pallidum and medial temporal lobe, we were not able to determine whether CBD had effects in other areas involved in emotional processing. Ideally, we would also have shown that effects of CBD on brain function were accompanied by effects on anxiety or psychotic symptoms. However, the study was powered to detect neural, as opposed to symptomatic effects. Future studies in larger samples are therefore required to investigate effects on symptoms. In addition, while we demonstrated that CBD has effects on the striatum and medial temporal cortex, whether these effects are mechanistically related to its antipsychotic or even anxiolytic effects remains unclear, as we did not examine these in the present study. This study also only reports on the acute effects of CBD, and it is possible that the effects may differ after a sustained period of treatment. It could also be argued that a parallel group of healthy controls receiving CBD would have helped to disentangle potential placebo effects. However, the healthy control group in the current study was primarily included to help determine whether the effects of CBD on brain activation were localised to those regions where CHR patients under placebo conditions showed dysfunction compared to controls and whether the effect direction was consistent with normalisation of brain function. Absence of group differences in task performance may arguably be considered as a limitation of the present study. It is worth noting that the fMRI paradigm that we employed did not involve an explicit measurement of accuracy of fear perception. Instead, participants were instructed to indicate (via button press) the gender of the faces (expressing different levels of fear), thus involving the implicit processing of fearful faces. The behavioural task data (gender discrimination accuracy and reaction times) therefore indexed a general measure of participants’ attention to the task, as well as the extent to which the underlying emotional valence (fearful stimuli) modulated the accuracy of appraisal of gender, and were not significantly different between groups. This was because the study was designed to investigate group differences in brain activation (neurophysiological response) while processing fearful facial stimuli rather than in task performance (behavioural response) and was powered as such. Absence of significant group difference in task performance does not preclude significant group difference in neurophysiological response^92^ and may even be desirable, as it minimises the risk of group differences in neurophysiological response being a non-specific consequence of differences in task performance^93^. Therefore, the differences that we observed in brain function may be argued to be not confounded by an effect of differences between groups in performance levels. Nevertheless, one cannot underestimate the merits of using an fMRI task that can also probe performance differences in the accuracy of fear perception, which warrants investigation in appropriately designed future studies. In terms of our patient group, we recruited a representative sample of CHR individuals as typically found in specialist CHR services^94^. However, CHR populations are clinically heterogeneous and it therefore remains possible that our results would differ in samples stratified, for example, by the three component subgroups of the CAARMS. Such an investigation would, however, require significantly larger sample sizes, which will likely be achieved only through the future use of large multi-centre studies. Finally, it may also be argued that statistically non-significant numerical group differences in THC-positive urine drug screen results between the CHR groups may have affected the differences in brain activation that we detected between the placebo treatment vs the CBD group. It is worth noting that all CHR participants satisfied the diagnosis of CHR state irrespective of whether they tested positive or negative on urine drug screen tests on the study day. All participants were advised to abstain from using cannabis for 96 h and confirmed as such verbally on the study day and yet tested positive on urine drug screen, reflecting the longer elimination period of THC and its metabolites in urine in cannabis users^95^. However, none of the participants were clinically intoxicated at the time of presenting on the morning of the study day and clearly were not so by the time of their fMRI scanning, which occurred around 3–4 h later. Therefore, in our view it is very unlikely that group differences in urine positive CHR individuals would have had a substantial effect on our results in the absence of clinically evident intoxication in urine positive individuals and the small numbers who tested positive per CHR treatment group. Nevertheless, we cannot be absolutely certain that group differences in the numbers of CHR participants who tested positive for THC on urine drug screen, although not statistically significant, did not affect group differences (CHR-PLB vs CHR-CBD) in brain activation that we detected.

Despite these limitations, this study is the first to demonstrate that a single dose of CBD modulates activation of the medial temporal cortex and striatum during fear processing in CHR patients. In showing that CBD modulates function of the neural circuitry directly implicated in psychosis onset^23,74^, these results add to previous evidence that CBD may be a promising novel therapeutic for patients at CHR^19,66,96^. Our results also support further investigation of the potential utility of CBD outside of the CHR field in other populations, such as in those with anxiety.
